# Carcinoma of the Colon in an Adult with Intestinal Malrotation

**DOI:** 10.1155/2013/525081

**Published:** 2013-07-29

**Authors:** Michael Donaire, James Mariadason, Daniel Stephens, Sitaram Pillarisetty, Marc K. Wallack

**Affiliations:** Department of Surgery, Metropolitan Hospital, New York Medical College, USA

## Abstract

Colon cancer is the third most common cancer in the USA. Intestinal malrotation diagnosed in adulthood was, until recently, a very rare phenomenon. While patients may present with intestinal obstruction or abdominal pain, the diagnosis is now often made as an incidental finding by computed tomography (CT). Surprisingly we found only seven case reports of carcinoma of the colon in patients with malrotation; CT failed to make the preoperative diagnosis in a majority. Laparoscopic colon surgery is rapidly becoming standard of care for colon cancer. We present a case of carcinoma of the colon in an adult that thwarted attempts at laparoscopic resection due to failure to recognize malrotation preoperatively. The literature is reviewed, and the implications of malrotation in patients with colon cancer are examined.

## 1. Introduction

Intestinal malrotation is a congenital anomaly that generally presents in the first month of life. Until a spate of recent reports, adult malrotation was considered extremely rare. Carcinoma of the colon, on the other hand, is the second most common cancer. Surprisingly reports of carcinoma of the colon in adults with malrotation are so rare that we found only 7 case reports in the literature. We present our case and discuss the implications.

## 2. Case Report

A 52-year-old male was admitted to an outside institution with lethargy, weight loss of 30 kg, and severe unexplained anemia (hemoglobin 4.5 g/dL; hematocrit 15%). The patient felt better after transfusion of 4 units of packed red blood cells, and gastroscopy performed at the time revealed a healed duodenal ulcer and erosive gastritis.

**Figure 1:**
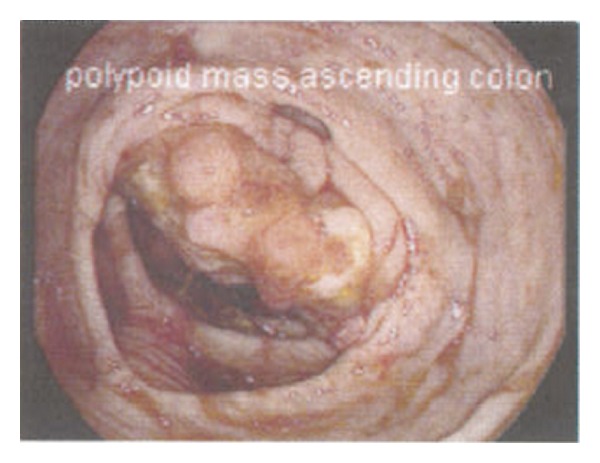
Right colon mass on colonoscopy.

**Figure 2:**
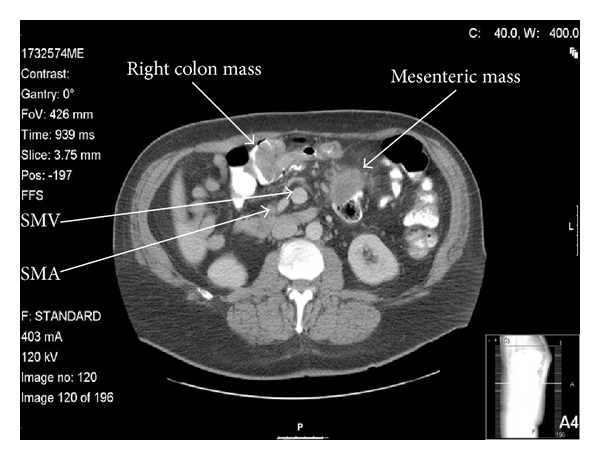
Right colon and mesenteric masses on CT.

**Figure 3:**
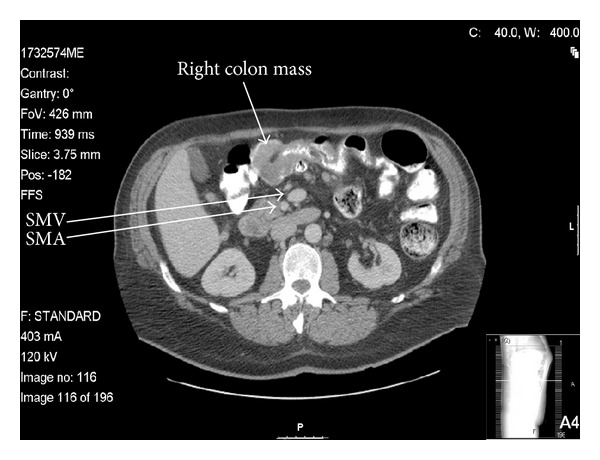
CT showing SMA dorsal to SMV.

When he lost his medical insurance, he was discharged and advised to have further workup performed elsewhere. During a difficult colonoscopy at a charity clinic, a large tumor was found in his right colon that precluded passage of the scope to the cecum (see Figure 1). Biopsy confirmed an infiltrating adenocarcinoma. Polyps in the sigmoid and transverse colon were also removed and found to be tubular adenomata. The patient was then referred to our institution, a safety-net hospital, where a CT scan was performed. The imaging demonstrated a 5 × 5 cm mass in the mesentery with spiculated calcifications, as well as an additional mass near the ileocecal valve that had the appearance of an intussusception (see Figure 2). Malrotation was not suspected, although later review of the imaging with a specialized CT radiologist demonstrated inversion of the normal SMA to SMV configuration (see Figure 3). Malrotation was not suspected. The liver was free of metastases. His past medical history included no prior surgery.

**Figure 4:**
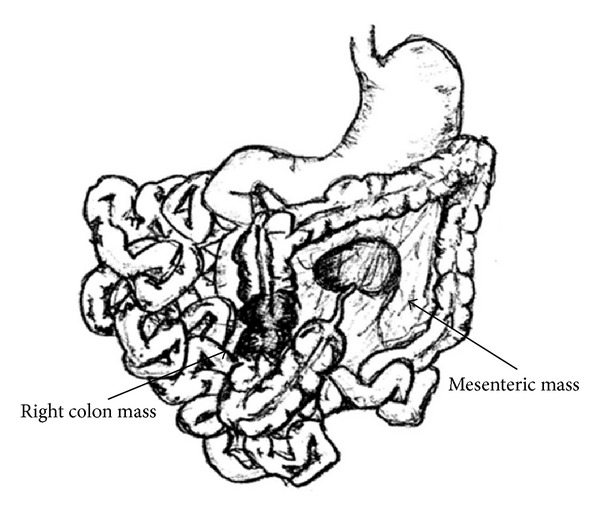
Depiction of intraoperative findings of malrotation and right colon and mesenteric masses.

**Figure 5:**
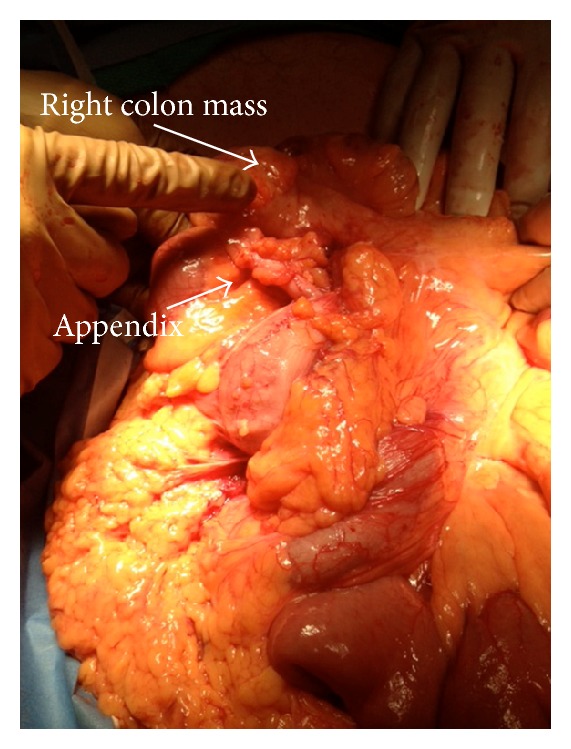
Intraoperative finding of right colon mass in setting of malrotation.

A laparoscopic right hemicolectomy was scheduled and commenced with introduction of a 10 mm optical trocar and two 5 mm ports. Upon entry, most of the small bowel was found plastered to the right flank, and the right colon was not visible. The left colon was visualized, but even after releasing adhesive bands holding the small bowel to the right side and mobilizing these loops to the left of midline, the ascending colon was not visible. It was decided that laparotomy was required to elucidate the findings. The abdomen was entered through a midline incision, and after mobilizing and packing off the small bowel to the left, the right paracolic gutter was found to be empty. The duodenojejunal junction, including the entirety of the duodenum, lays to the right of the vertebral column. The right colon was tethered to the side wall of the abdomen on the right by long bands (Ladd's bands), which were eventually divided to obtain adequate mobilization. The cecum and appendix were found to occupy the left upper quadrant (see Figures 4 and 5). A tumor was palpated in the ascending colon, and a firm mass in the mesentery to its left was thought to be metastatic nodes. The superior mesenteric vessels appeared to have their usual orientation. The right colon was thereby mobilized beyond the left branch of the middle colic, and a right hemicolectomy was completed. The patient made an uneventful recovery. 

Pathology revealed a 5 cm infiltrating adenocarcinoma of the ascending colon and metastatic tumor in the mesentery with no evidence of lymphatic tissue involvement. 23 lymph nodes removed were negative for metastases. The patient received chemotherapy and is doing well 12 months after surgery.

## 3. Discussion

Intestinal malrotation, a rare congenital disorder occurring in about 1 in 6,000 live births, results from incomplete rotation and fixation during fetal development. Rapid differential growth of the midgut, starting at gestational week five with herniation into the proximal portion of the umbilical cord, is normally followed by a 270-degree counterclockwise rotation around the superior mesenteric artery (SMA) as the intestine returns to the abdomen at week ten and fixes to the retroperitoneum [1]. Arrest of development anywhere along this process results in malrotation, which may be classified as reversed rotation, nonrotation, or varying degrees of rotation. In reversed rotation, there is an abnormal 90-degree clockwise rotation of the midgut with the cecum lying to the right of and dorsal to the SMA [2]. Nonrotation is the complete failure of midgut rotation around the SMA, whereby the duodenojejunal segment is ultimately confined to the right and the large intestine largely to the left hemiabdomen. Our patient would seem to have had a form of nonrotation. Balthazar further classified malrotation based on involvement of duodenojejunal loops, ileocolic loops, or both [3]. 

Malrotation is diagnosed during the first month of life in 85–90% of cases; its presentation in adults, therefore, has traditionally been considered extremely rare. Symptomatology ranges from the asymptomatic to nebulous postprandial pain to complete obstruction, which compounds the difficulty in making the diagnosis and recognizing the true incidence of malrotation [4]. However, with increased use of advanced imaging, the diagnosis is being made more frequently in adults. A study of barium enemas on 2000 adults demonstrated a prevalence of 0.2% malrotation [5]. In a more recent report, however, 48% of 170 malrotation cases from a single institution were adults [6]. 

Colon cancer is statistically the third most common cancer in the U.S. It is therefore surprising that colon cancer occurring in adults with malrotation has not been reported more often. In a search of the literature, only 7 other cases of carcinoma of the colon in patients with malrotation were located [7–13]. None of the reported cases were from North America.

Even with the proliferative use of CT imaging, the diagnosis of malrotation in adults may be missed unless a high index of suspicion exists. The imaging modality of choice is the upper gastrointestinal series, which has a sensitivity of 80%. It may demonstrate an abnormal position of the duodenojejunal junction on the right side of the abdomen better than CT [14]. Ultrasonography may, furthermore, exhibit the reversal of normal superior mesenteric vessel orientation. The challenges in diagnosing appendicitis or volvulus in malrotation cases have also been well documented [7, 15]. CT scans are now the most frequently used modality for the diagnosis of malrotation. While the modality was employed in 4 of the 7 reported cases of colon carcinoma with adult malrotation, CT failed to demonstrate malrotation in 3 of 4 instances [8–11]. This mirrored our experience with the presented patient. Lymphatic mapping and angiography were used by 2 authors, which allowed for a preoperative diagnosis. 

As in our patient, 5 of the 7 reported cases arose from the right colon. In this situation, right hemicolectomy obviates the need for a Ladd's procedure, but may need to be considered for left sided cancer. One case of laparoscopic right hemicolectomy for cecal carcinoma, concomitant with malrotation, was described recently [13]. When the finding is a surprise during planned laparoscopic right hemicolectomy, failure to locate the right colon with the laparoscope may precipitate conversion to open surgery, as was our experience. 

## 4. Conclusion

Carcinoma of the colon occurring in patients with adult malrotation is extremely rare with only a handful of cases reported. The preoperative recognition of malrotation would allow for better surgical planning and possible successful completion of a laparoscopic resection. Yet the preoperative diagnosis of malrotation has been relatively rare despite the use of CT. Greater awareness of the possibility of malrotation and use of contrast studies like upper gastrointestinal series and barium enemas may help to elucidate the diagnosis in some cases of colon cancer.
